# The applicability of current global cardiovascular risk scores and cardiovascular surrogates in chronic obstructive pulmonary disease

**DOI:** 10.1186/1532-429X-18-S1-P134

**Published:** 2016-01-27

**Authors:** Mohammed Y Khanji, Ian S Stone, Wai-Yee James, Armida Balawon, Leonette John, Redha Boubertakh, Neil C Barnes, Steffen E Petersen

**Affiliations:** 1 Queen Mary University London/ Barts Health NHS Trust, Center for Advanced Cardiovascular Imaging and Research, London, UK; 2 grid.139534.90000000103725777Department of Respiratory Medicine, Barts Health NHS Trust, London, UK; 3 Global Respiratory Franchise, GlaxoSmithKline, London, UK

## Background

Chronic obstructive pulmonary disease (COPD) is a complex disorder associated with significant cardiovascular morbidity and mortality. Despite this, current cardiovascular scoring systems do not include COPD in their risk prediction models. The aims of this case- control study were to assess whether differences in cardiovascular surrogate markers exist in COPD to further understand the relationship of COPD to cardiovascular structure and function.

## Methods

We performed a post-hoc cross-sectional analysis utilising baseline data from two randomized controlled trials (n = 36 and 54). 26 COPD patients with lung hyperinflation were matched for global cardiovascular risk, using the UK population validated QRISK2 score, with 26 controls with normal lung function. Patients underwent 1.5T cardiac magnetic resonance imaging, non-invasive arterial stiffness assessment and lung function testing.

## Results

Cardiac chamber size and stroke volume was decreased in COPD patients with lung hyperinflation compared to controls matched for cardiovascular risk. The mean differences in left ventricle stroke volume index (LVSVI), left and right ventricular end diastolic volume index were -10.3 ml/m2 (95% CI -15.1, -5.5, p < 0.001), -14.1 ml/m2 (95% CI -21.9,-6.3 p < 0.001) and -13.0 ml/m2 (95% CI -23.3, -2.6 P < 0.015) respectively, which were shown to be associated with airflow limitation in multivariate models.

Pulse wave velocity (PWV) (mean difference +1.0 m/s, 95% CI 0.02-1.92; p = 0.045) and total arterial compliance (TAC) (mean difference -0.27 mL/m2/mmHg, 95% CI -0.39, -0.15; p < 0.001) were adversely affected in COPD compared to the control group. In the whole cohort (n = 90) QRISK2 (β= 0.046, p = 0.017) and FEV1 (β=-0.013, p = 0.022) were associated with PWV in multivariate analysis. The relationship between QRISK2 and PWV appeared to be modified by COPD, where the interaction term reached borderline significance (p = 0.060). FEV1 (β=0.005, p = 0.004) was also associated with TAC in multivariate analysis.

## Conclusions

Surrogate markers for cardiovascular outcomes are adversely affected in COPD compared to a group matched for global cardiovascular risk with normal lung function. Our findings suggesting that current scoring systems may be suboptimal for cardiovascular risk prediction in COPD.

**Figure 1:**
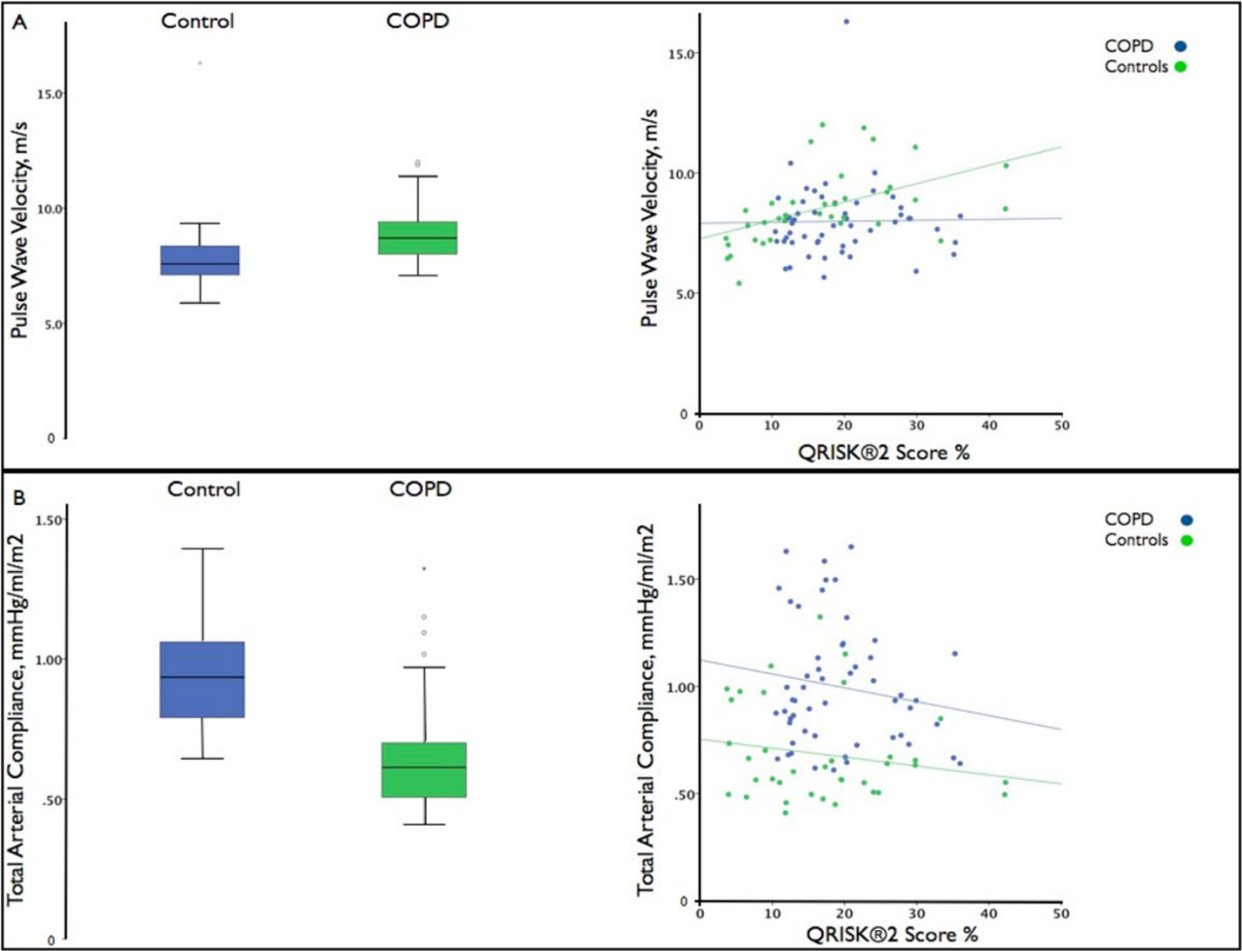
**Differences in PWV, TAC and their relationship to QRISK2 in COPD compared to controls matched for cardiovascular risk**.

